# Investigation of phytotherapeutic potential of herbal mixtures and their effects on salbutamol induced cardiotoxicity and hyperlipidemia in rabbits

**DOI:** 10.1186/s40529-023-00394-9

**Published:** 2023-07-19

**Authors:** Nida Liaqat, Nazish Jahan, Khalil ur Rahman, Iqra Tahseen, Tauseef Anwar, Huma Qureshi

**Affiliations:** 1 Department of Chemistry, University of Agriculture, Faisalabad, 38000 Pakistan; 2 Department of Chemistry, Rawalpindi Women University, Rawalpindi, 46300 Pakistan; 3 Department of Biochemistry, University of Agriculture Faisalabad, Faisalabad, 38000 Pakistan; 4 Department of Botany, The Islamia University of Bahawalpur (Baghdad ul Jadeed Campus), Bahawalpur, 6300 Pakistan; 5 Department of Botany, University of Chakwal, Chakwal, 48800 Pakistan

**Keywords:** Medicinal potential, *Coriandrum sativum*, *Piper nigrum*, *Cactus grandiflorus*, Salbutamol, Cardiac markers

## Abstract

**Background:**

Cardiovascular diseases (CVDs) are the major cause of deaths all over the world. The high level of blood cholesterol and oxidative stress are major risk factors for heart diseases. The phytotherapeutics have attracted attention as potential agents for preventing and treating oxidative stress associated diseases. The objective of present study was to evaluate the synergetic cardio-protective and antilipidemic potential of medicinal plants viz. *Coriandrum sativum*, *Piper nigrum* and *Cactus grandiflorus*. Cardio-protective and anti-lipidemic potential of herbal mixture was evaluated against salbutamol induced cardiotoxicity in rabbits. For this purpose, rabbits were divided into six groups as normal control, salbutamol control, curative and standard drug curative.

**Results:**

Salbutamol significantly (p < 0.05) increased the level of serum cardiac biomarkers (ALT, CK-MB, AST and LDH) and lipids (LDL, triglycerides, cholesterol) in rabbits. The prior and post administration of herbal mixture significantly (p < 0.05) lowered the elevated level of serum cardiac biomarkers and lipids equal to normal control. Gross pathological examination revealed that heart of salbutamol control animals became hardened, congested and were enlarged than preventive and curative groups. The phytotherapeutic analysis of medicinal plants revealed the presence of phenols, tannins, alkaloids and steroids.

**Conclusion:**

The results showed that this herbal mixture has strong cardio-protective and anti-lipidemic potential.

**Supplementary Information:**

The online version contains supplementary material available at 10.1186/s40529-023-00394-9.

## Background

Cardiovascular diseases (CVDs) are group of diseases including rheumatic heart disease, peripheral arterial disease, ischemic heart disease and congenital heart diseases. According to recent studies, CVDs account for 30% of global deaths (Silwa et al. 2008; Abbasi et al. 2013). According to National Health Service Professionals (NHSP-UK), death rate due to CVDs and hypertension is 17.9%. The CVDs rate in South Asians like Pakistan is probably to be double in next 20 years (Saleheen et al. 2009). High risk of cardiac diseases is associated with high levels of serum Low-density lipoprotein (LDL) and low levels of high-density lipoprotein (HDL). Hyperlipidemia leads to vulnerable atherosclerotic plaque followed by occlusion of a coronary artery (Jahan et al. 2012; Nelson 2013). The increased production of free radicals is a big risk factor for heart diseases (Oguntibeju et al. 2010). The use of natural antioxidants may decrease cellular injury and apoptosis through a radical-scavenging mechanism and is promising therapy among pharmacological interventions to protect heart against oxidative stress (Patel et al. 2007). Lowering of cholesterol level and inhibition of free radical formation are proved effective strategies to reduce the risk of cardiac diseases. Phenolic compounds have attracted attention as potential agents for treating oxidative stress associated diseases (Gan et al. 2010; Rivas-Arreola et al. 2010). Developing countries, like Pakistan are not able to control and provide the successful solution for the management and treatment of cardiac disorders. Plant based drugs play dynamic role in the management and treatment of cardiovascular disorders. Many studies have shown medicinal plants useful in protection of chemically induced cardiotoxicity (Zarei et al. 2013; Shatoor et al. 2014).

Some medicinal plants are reported for cardio-protective potential but limited reports are available on combinations of medicinal plants. Plants when used in combination may show better potential because of synergetic effect. It is therefore preferable to use herbal combinations instead of depending on single herb. Combination of following three medicinal plants was selected to study the cardio-protective potential. Pharmacological studies of *Coriandrum sativum* (vern. dhanya) have confirmed its hypolipidemic, hypoglycemic, anti-mutagenic, antimicrobial, antioxidant and antihypertensive activity (Duman et al. 2010). *Cactus grandifloras* is a good source of polyphenols. No sufficient work has been done revealing a variety of pharmacological effects of this plant especially in cardiovascular diseases and cancer. *Piper nigrum* (vern. kali mirch) has antimicrobial, antimutagenic, antioxidant and radical scavenging property (Gulsin 2005; Vijayakumar et al. 2004). With all these wide range of medicinal properties, the objective of present study is to evaluate the synergism of anti-lipidemic and cardio-protective potential of combination of three medicinal plants in chemically induced cardiotoxicity and hyperlipidemia.

## Methods

### Selection of plants

*Coriandrum sativum*, *Piper nigrum* and *Cactus grandiflorus* were selected for phytotherapeutic (phytochemical) analysis and cardio-protective activity. Seeds of *C. sativum* and *P. nigrum* were collected from local market of Faisalabad. While alcoholic extract of *Cactus grandiflorus* (fruit) was purchased from local chemical store.

### Preparation of plant extracts

The plant material coriander and pepper seeds were ground to powdered form. Powdered plant material (30 g) of coriander seeds and pepper was defatted separately with 150 ml hexane by reflux method for half an hour. After that, the mixture was filtered and the residue was refluxed with 200 ml methanol. After refluxing, methanolic extract was filtered through Whatman No.1 filter paper and concentrated at reduced pressure by rotary evaporator.

### Phytochemical profile

#### Qualitative estimation of phytochemicals

The detection of phytochemicals in *C. sativum* (seeds), *P. nigrum* (seeds) and *C. grandiflorus* (fruit) was done by using following methods (Usman et al. 2009; Raja 2012; Tamilselvi et al. 2012).

##### Alkaloids

small quantity of the extract was stirred with 5 ml of 1% aqueous HCl on water bath and then filtered. Of the filtrate, 1 ml was taken individually into 2 test tubes. To the first portion, few drops of Dragendorffs reagent was added; occurrence of orange-red precipitate was taken as positive. To the second, 1 ml Mayer’s reagent was added and appearance of buff-colored precipitate was taken as indication for the presence of alkaloids.

##### Flavonoids

Small quantity of the extract was dissolved in water and filtered. To 5 ml of each of the filtrate, 3 ml of lead ethanoate solution was then added. Appearance of a buff-colored precipitate indicated the presence of flavonoids.

##### Saponins

One gram of each portion was boiled with 5 ml of distilled water and filtered. To the filtrate, about 3 ml of distilled water was further added and shaken vigorously for about 5 min. Frothing which persisted on warming was taken as evidence for the presence of saponins.

##### Terpenoids

A little of each portion was dissolved in ethanol. To it 1 ml of acetic anhydride was added followed by the addition of conc. H_2_SO_4_. A change in color from pink to violet showed the presence of terpenoids.

##### Steroids

To 0.2 g of each portion, 2 ml of acetic acid was added; the solution was cooled well in ice followed by the addition of conc. H_2_SO_4_ carefully. Color change from violet to blue or bluish-green indicated the presence of a steroidal ring i.e., aglycone portion of cardiac glycoside.

#### Quantitative estimation of phytochemicals

##### Alkaloid quantification

Alkaloid quantification was performed by Harborne method (Harborne 1973). The percentage yield of alkaloids was calculated by following formula.

$$\displaylines{\% age{\text{ }}of{\text{ }}alkaloid = \cr \frac{{Amount{\text{ }}of{\text{ }}Alkaloid{\text{ }}obtained}}{{Amount{\text{ }}of{\text{ }}dry{\text{ }}sample{\text{ }}used}} \times 100 \cr}$$

##### Saponin quantification

The quantification of saponin was done by using method described by Obadoni and Ochuko (2001). The percentage yield of saponin was calculated by using formula.

$$\displaylines{\% age{\text{ }}of{\text{ }}saponin = \cr \frac{{Amount{\text{ }}of{\text{ }}saponin{\text{ }}obtained}}{{Amount{\text{ }}of{\text{ }}dry{\text{ }}sample{\text{ }}used}} \times 100 \cr}$$

##### Determination of total phenolic contents

Modified Folin-Ciocalteu method was used to determine the total phenolic contents of plants material (Aslam et al. 2012). Gallic acid was used as standard compound to compare results. Total phenolic contents of plant extract were determined by the following formula and results were expressed as mg of gallic acid equivalent per gram of plant extract.

$$T = C \times V/M$$

Where; T = Total phenolic contents in mg GAE/g of plant extract; C = the unknown concentration of plant extract calculated from standard curve in mg/ml; V = the volume of the extract taken in ml; M = the weight of plant extract taken in grams.

##### Determination of total tannins

The tannins were determined by Folin and Ciocalteu method by some modification (Tamilselvi et al. 2012). Tannic acid was used as standard compound to compare results. The results of tannins were expressed in terms of tannic acid mg/g of extract.

$$T = TA \times V/M$$

Where; T = Total Tannic acid contents in mg/g of plant extract; TA = the unknown concentration of plant extract calculated from standard curve in mg/ml; V = the volume of the extract taken in ml; M = the weight of plant extract taken in grams.

##### Determination of total flavonoid contents

Total flavonoid content was determined using aluminum chloride (AlCl_3_) method followed by (Eghdami and Sadeghi 2010). Catechin was used as a standard. The results were expressed as mg catechin (CE)/g of plant extract.

$$T = CE \times V/M$$

Where; T = Total flavonoid contents in mg/g of plant extract; CE = the unknown concentration of plant extract calculated from standard curve in mg/ml; V = the volume of the extract taken in ml; M = the weight of plant extract taken in gram.

### Cardioprotective and antilipidemic activity

#### Experimental protocol

Cardioprotective and antilipidemic potential of herbal mixture was evaluated in chemically induced myocardial intoxication in animal model. Herbal mixture was consisting of *C. sativum* seeds, *P. nigrum* seeds and *C. grandiflorus* fruit in 1:2:1 ratio respectively. Rabbits were kept for a week accommodation period and then divided into six groups; each group consisting of three rabbits (Table 1). Hyperlipidemia was induced by using high hyperlipidemic diet including coconut oil, dry milk, vegetative ghee and butter.

**Table 1:** Treatment detail in experimental groups of rabbits

Group no.	Detail
1	**Normal control**: only standard diet was given to animals.
2	**Salbutamol control**: rabbits were treated with salbutamol (65 mg/Kg) for consecutive two days. The blood sample was collected daily.
3	**Baseline group**: rabbits were treated with herbal mixture 100 mg/kg once daily for three weeks. The blood sample was collected.
4	**Preventive group**: rabbits were treated with herbal mixture 100 mg/kg and then with salbutamol (65 mg/Kg) for two consecutive days. The blood sample was collected daily.
5	**Curative group**: to induce cardiotoxicity, salbutamol (65 mg/kg) was given to rabbits for two days. Then these myocardial infracted rabbits were treated with 500 mg/kg of herbal mixture once daily for five days and blood samples were collected daily.
6	**Standard drug curative group**: rabbits were treated with salbutamol (65 mg/kg) for two days to induce cardiotoxicity. Then these cardio infarcted rabbits were treated with mixture of three standard drugs including Captopril (25 mg), Amlodipine Besylate (5 mg) and Atenolol (25 mg) on a daily basis for five days and blood samples were collected daily.

#### Collection of blood

The blood sample were collected in centrifugation glass tubes, then centrifuged the blood and collected the separated serum, and stored in a deep freezer for biochemical analysis.

### Biochemical assessment

#### Cardiac profile

At the end of experiment different cardiac biomarkers such as ALT, LDH, AST, CK-MB was analyzed by using chemical bio-analyzer with commercially available kits.

#### Lipid profile

The activity of serum lipids, cholesterol, HDL, LDL, triglycerides was determined by kit method.

#### Gross pathological examination

At the end of the experiment, rabbits were slaughtered and gross pathology was performed with the help of veterinary doctor. Obvious changes in the weight and structure of the heart, kidneys, liver and lungs were noted.

### Statistical analysis

All samples were examined in triplicate and data were expressed as mean ± SEM. Data was analyzed using analysis of variance (ANOVA) in SPSS 21 software. Tukey’s multiple comparison test was used for comparison of means of different treatments (p < 0.05).

## Results

### Phytochemical screening

Phytotherapeutical screening of plants revealed the presence of phytoconstituents like alkaloids, flavonoids, tannins and saponins in *C. sativum* (seeds), *P. nigrum* (seeds) and *C. grandiflorus* (fruits). Quantitative estimation of the percentage crude alkaloid and saponin in these medicinal plants is summarized in Table 2. Coriander seeds had highest alkaloid percentage while *P. nigrum* had highest saponin percentage.

**Table 2:** Phytochemicals screening of methanolic extracts of the *Coriandrum sativum*, *Piper nigrum* and *Cactus grandiflorus*

Phytochemicals	*Coriandrum sativum*	*Piper nigrum*	*Cactus grandiflorus*
**Alkaloids**	+ (4.31 ± 0.06)	+ (3.2 ± 0.1)	- (0.04 ± 0)
**Flavonoids**	+ (41.4±0.58)	- (3.7 ± 0.1685)	- (3.98 ± 0.35)
**Saponins**	+ (4.7±10).	+ (5.4 ± 0.36)	- (0.00 ± 0)
**Terpenoids**	+ (48.23± 0.76)	- (37.51 ± 1.38)	- (34.68 ± 2.33)

+ Presence - Absence

### Determination of total phenolics, tannins, flavonoids contents

Different methods have been used to find out the phenolics, tannins and flavonoid contents of medicinal plants. *P. nigrum* showed highest total phenolic and flavonoid contents while Coriander (seeds) showed highest total tannins contents (Table 2). Data is represented as mean ± SEM (n = 3).

### Cardioprotective and antilipidemic potential

The cardio protective and antilipidemic potential of medicinal plants was evaluated through preventive and curative mode of treatment.

#### Preventive effect

Rabbits were pre-treated with herbal mixture for 20 days and then treated with Salbutamol (65 mg/ kg) once a day for two days to induce myocardial injury. Salbutamol significantly (P < 0.05) increased the level of serum cardiac markers (ALT, CK-MB, AST, LDH) and lipids (LDL, cholesterol, triglycerides) in salbutamol-induced group than normal control group showing myocardial infarction in rabbits. HDL level was decreased significantly in salbutamol group as compared to normal control group. Prior administration of herbal mixture (100 mg/kg) significantly reduced the salbutamol induced increased level of cardiac biomarkers and lipids (Table 3).

**Table 3:** Preventive effect of herbal mixture on cardiac biomarkers and lipids level (1U/L)

Enzymes Parameters	Normal Control	Salbutamol Control	Baseline	Preventive
**CK-MB**	50.53 ± 0.33	91.3^*^ ±0.047	70.45 ± 0.81	47.39^**^ ±0.39
**LDH**	347.2 ± 0.42	699.46^*^ ±0.098	473.2 ± 0.039	409.67^**^±0.27
**AST**	46.33 ± 0.49	117.7^*^ ±1.42	43.46 ± 0.12	65.9^**^±1.03
**ALT**	48 ± 0.3	130.62^*^ ±0.47	59.24 ± 0.125	24.82^**^±0.85
**LDL**	79.6 ± 1.02	159.4^*^±2.7	32.0 ± 1.34	78.0^**^±1.5
**HDL**	85.0 ± 0.667	41.5^*^ ±0.55	42.2 ± 0.577	95.0^**^±1.75
**Triglycerides**	175.0 ± 0.4	335.2^*^ ±1.42	232.5 ± 0.318	195.5^**^±0.45
**Cholesterol**	160.3 ± 1.7	142.0^*^ ±0.753	58.24 ± 1.4	62.5^**^±0.235

Results are expressed as mean ± SEM for 3 rabbits in each group

*Significantly different from control group (P < 0.05)

**Significantly different from salbutamol control group (P < 0.05)

#### Curative effect

In curative cardio-protective activity, cardiotoxicity was induced in rabbits and then herbal mixture (500 mg/kg) was given to myocardial infarcted rabbits once daily for five days. Salbutamol significantly increased (P < 0.05) the level of diagnostic cardiac biomarkers and lipids in serum of the rabbits as compared to the rabbits of normal control group showing myocardial infarction (Table 4). But post administration of herbal mixture significantly (P < 0.05) reduced the elevated level of enzymes and come close to normal group. These results are comparable with the effect of standard drug. Combination of three standard drugs including Captopril (25 mg), Amlodipine Besylate (5 mg) and Atenolol (25 mg) was used to compare the activity of medicinal plants. The baseline content of these enzymes was found to be normal which revealed that herbal mixture at a dose 500 mg/ kg did not induces any cardio-toxic effects.

**Table 4:** Curative effect of herbal mixture on cardiac markers and lipids level (1U/L)

Cardiac Marker Enzymes	Days	Normal Control	Salbutamol Control	Curative	Standard Drug
**CK-MB**	1	50.2 ± 0.26	92.1 ± 1.32^*^	62.4 ± 2.48^**^	61.33 ± 2.50^*^
	2	50.9 ± 1.68	90.5 ± 1.29^*^	69.3 ± 1.69^**^	54.67 ± 1.22^*^
	3	51.12 ± 2.34	89 ± 0.76^*^	62.44 ± 2.68^**^	53.8 ± 1.24^*^
	4	51.14 ± 0.98	86.7 ± 0.98^*^	56 ± 4.54^**^	52 ± 0.98^*^
	5	51.26 ± 3.24	85 ± 1.45^*^	53.7 ± 1.11^**^	52.3 ± 1.04^**^
**LDH**	1	347.2 ± 0.066	689.3 ± 0.34^*^	479.8 ± 0.63^**^	465.1 ± 0.03^*^
	2	347.58 ± 0.13	699.4 ± 1.23^*^	472.5 ± 0.33^**^	448.3 ± 0.55^*^
	3	347.93 ± 1.05	698.8 ± 3.24^*^	473.1 ± 0.30^**^	432.6 ± 0.02^*^
	4	348.01 ± 0.47	697.4 ± 2.18^*^	473.05 ± 0.12^**^	422.12 ± 0.79^*^
	5	348 ± 0.99	705.4 ± 0.97^*^	473 ± 0.69^**^	420.4 ± 0.67^**^
**AST**	1	42.5 ± 0.73	118.4 ± 1.47^*^	73.7 ± 1.45^**^	63.2 ± 1.99^*^
	2	43.25 ± 0.12	119.28 ± 1.84^*^	68 ± 0.97^**^	62.1 ± 1.87^*^
	3	44.12 ± 0.35	120.3 ± 0.06^*^	56.2 ± 1.33^**^	60.6 ± 1.28^*^
	4	44.45 ± 0.53	122.9 ± 0.78^*^	54.5 ± 0.09^**^	59 ± 0.33^**^
	5	44.23 ± 0.09	124.7 ± 0.99^*^	54 ± 0.48^**^	59.07 ± 2.54^*^
**ALT**	1	46.7 ± 1.07	130 ± 1.18^*^	89.2 ± 1.22^**^	84.2 ± 1.39^*^
	2	47.42 ± 0.51	133 ± 2.48^*^	86.6 ± 1.35^**^	71.9 ± 0.01^*^
	3	47.39 ± 0.01	134.8 ± 0.04^*^	79.6 ± 2.31^**^	69.67 ± 0.82^*^
	4	48.22 ± 1.27	135.6 ± 1.05^*^	72.3 ± 0.03^**^	69.54 ± 0.46^*^
	5	48.44 ± 1.39	140.4 ± 0.02^*^	70.1 ± 0.29^**^	68.22 ± 1.27^*^
**LDL**	1	26.0 ± 1.75	60.7 ± 2.31^*^	50 ± 1.75^**^	57.7 ± 2.35^*^
	2	25.7 ± 1.34	62 ± 2.1^*^	45 ± 1.5^**^	53.6 ± 2.30^*^
	3	26.2 ± 1.25	65 ± 2.2^*^	40 ± 0.79^**^	45.4 ± 2.15^*^
	4	24.7 ± 0.75	70 ± 2.75^*^	35 ± 1.83^**^	35 ± 0.85^*^
	5	25.3 ± 1.01	72 ± 2.9^*^	30 ± 1.11^**^	30.9 ± 1.04^**^
**HDL**	1	79.0 ± 1.066	30.1 ± 1.34^*^	87.3 ± 1.63^**^	69.1 ± 1.03^*^
	2	83.2 ± 2.13	33.2 ± 1.23^*^	88.5 ± 2.33^**^	88.2 ± 0.55^*^
	3	81.5 ± 1.85	32.7 ± 1.24^*^	90.2 ± 1.30^**^	85.2 ± 0.02^*^
	4	85.67 ± 1.47	33.5 ± 2.18^*^	95.3 ± 1.12^**^	93.12 ± 0.79^*^
	5	79.9 ± 1.99	31.4 ± 1.97^*^	86.3 ± 2.69^**^	81.4 ± 0.67^**^
**Triglycerides**	1	221.2 ± 1.73	335.4 ± 2.47^*^	234.7 ± 1.45^**^	298.3 ± 1.99^*^
	2	221.5 ± 2.12	335.28 ± 1.84^*^	232 ± 2.97^**^	275.2 ± 1.87^*^
	3	223.7 ± 1.35	335.3 ± 2.06^*^	230.2 ± 1.23^**^	225.1 ± 1.28^*^
	4	220.47 ± 2.26	335.9 ± 3.78^*^	225.5 ± 0.79^**^	223.2 ± 2.33^**^
	5	222.40 ± 1.09	335.7 ± 2.99^*^	220 ± 1.48^**^	221.0 ± 2.54^*^
**Cholesterol**	1	60.7 ± 1.07	143.0 ± 1.18^*^	129.2 ± 3.22^**^	120.2 ± 3.15^*^
	2	61.42 ± 1.51	142.0 ± 2.48^*^	124.6 ± 3.35^**^	114.5 ± 2.7^*^
	3	62.39 ± 2.01	142.8 ± 2.04^*^	79.1 ± 2.31^**^	101.2 ± 2.5^*^
	4	62.22 ± 1.72	141.6 ± 2.05^*^	79.3 ± 1.03^**^	78.54 ± 1.46^*^
	5	61.44 ± 1.90	141.4 ± 1.02^*^	63.1 ± 2.29^**^	70.22 ± 0.70^*^

Results are expressed as mean ± SEM for 3 rabbits in each group

*significantly different from control group (P < 0.05)

**significantly different from salbutamol control group (P < 0.05)

### Gross pathology

Significant changes occurred in different organs of rabbits due to the administration of salbutamol. Heart was normal in all groups except salbutamol induced group. There was no considerable change observed in the kidneys of all groups except salbutamol group. Consistency of lungs was normal in all the groups. The pale color of normal control group was due to surroundings in which they kept. Weight of each organ was also noted. All the results are enlisted in Table 5.

**Table 5:** Gross pathological studies of different organs along weight (g) of rabbits

Organs	Heart	Liver	Lungs	Kidneys (R: Right; L: Left)
**Normal control**	Normal Consistency & color (3.5 g)	Consistency & color Normal (21.6 g)	Normal Consistency Paled in color (5.1 g)	Both kidneys normal (R: 4.5 g; L: 5.1 g)
**Salbutamol control**	Enlarged, Hardened, Necrosed, Congested (5.4 g)	Normal Consistency & color (35.2 g)	Normal Consistency (11.7 g)	Consistency normal but mild necrosis on right kidney (R: 5.4 g; L: 5.3 g)
**Baseline**	Normal Consistency & color (2.8 g)	Normal Consistency but friable (24.8 g)	Normal Consistency & color (7.9 g)	Both kidneys normal (R: 4.4 g; L: 4.1 g)
**Preventive**	Normal Consistency & color (2.7 g)	Normal Consistency (23.8 g)	Normal Consistency & color (7.8 g)	Normal Consistency (R: 4.2 g; L: 4.5 g)
**Curative**	Consistency normal (2.1 g)	Consistency normal Mild necrosed (33.7 g)	Consistency normal (5.8 g)	Both kidney normal (R: 5.4 g; L: 4.3 g)
**Standard drug**	Consistency normal Congested (3.1 g)	Consistency normal (34.1 g)	Consistency normal (9.5 g)	Consistency normal (R: 5.3 g; L: 5.1 g)

## Discussion

The present study demonstrated the phytotherapeutic profiling and cardio-protective and antilipidemic potential of medicinal plants. Phytochemicals, phenolic compounds are dietary constituents’ generally present in plants and have been considered to have high antioxidant and free radical scavenging ability (Kahkonen et al. 2001). Phenolic compounds are necessary for plants due to their reducing ability due to the presence of hydroxyl groups (Elmastas et al. 2006). Flavonoids can demonstrate their cardio-protective potential by different mechanisms. The intake of flavonoids can prevent endothelial dysfunction by elevating the vaso-relaxation process leading to decline of arterial pressure (Kurosawa et al. 2005). Flavonoids may directly scavenge some radical species and moreover assist in uptake of oxidative modified low-density lipoprotein (LDL) through scavenger receptors (Burns 2000).

### Effect of herbal mixture on serum cardiac profile

Salbutamol or albuterol is β_2_-adrenergic receptor agonist used for the aid of bronchospasm in conditions such as chronic obstructive pulmonary and asthma disease. A numeral study was observed on isoproterenol induced cardiotoxicity but on salbutamol induced myocardial infarction only few works has been done (Kousar et al. 2012). Because of structural resemblance of salbutamol with isoproterenol it is understood that its mode of action may be similar to isoproterenol.

Salbutamol significantly (P < 0.05) increased the level of serum cardiac marker enzymes like ALT, CK-MB, AST, LDH in salbutamol induced group as compared to normal control group indicating myocardial infarction in rabbits. The increase in enzyme level may be due to increased Ca^+ 2^ concentration in blood due to salbutamol as a result secretion of enzymes increases. The high levels of enzymes are a sign of the strictness of salbutamol induced myocardial cell necrosis. The myocardial cell necrosis can be due to increase in lipid peroxidation. Pre and Post administration of herbal mixture significantly decreased the salbutamol induced elevated level of cardiac marker enzymes. The decrease in enzymes levels could be due to potential of plants for repairing and protection of the membrane due to antioxidant polyphenols, thereby preventing the secretion of enzymes (Ojha et al. 2011; Qureshi et al. 2016).

### Effect of herbal mixture on lipid profile

Salbutamol significantly (P < 0.05) increased the level of cholesterol, LDL, triglycerides and decreased HDL in salbutamol group as compared to normal control group. However, the pretreatment with herbal mixture reduced the elevated level of cholesterol, LDL, triglycerides. Lipid peroxidation, due to excessive free radicals has been identified as one of the major destructive reactions in cellular mechanism of the myocardial infarction. Highly oxidative metabolite of salbutamol accelerates rate of peroxidation in membrane phospholipids and releases free fatty acids into plasma by the action of phospholipase A_2_ and it is a major causative factor of salbutamol induced hyperlipidemia (Sivakumar et al. 2007).

Mixture of *Coriandrum sativum*, *Piper nigrum* and *Cactus grandiflorus* demonstrated significant (P < 0.05) reduction in enzyme level when compared to salbutamol control group. This decline in enzyme level could be due to the action of herbal mixture on maintaining membrane consistency thus inhibited the steady flow of enzymes.

Herbal mixture shows cardio protective and antilipidemic activity. Individual plant has its own properties but when two or more plants/herbs mix together they will probably show more useful results against diseases. Some studies were reported the cardio protective activity of these individual plants. *C. sativum* seed has a potential to lessen threat of heart diseases by decreasing the elevated level of cardiac marker enzymes and by increasing the endogenous antioxidants like superoxide dismutase (Kousar et al. 2012). *C. sativum* seeds have β-carotene constituent responsible for antioxidant action (Guerra et al. 2005), which can be responsible for its cardio protective activity. *P. nigrum* has glycoside so it shows cardio protective function. *C. grandiflorus* is good source of polyphenols. It is thought that *Cactus* raises arteriolar pressure by increasing the muscular power of the heart and causing arteriolar contraction (Verma et al. 2012).

### Gross pathology

The necrosis on heart showed that some kind of disease induced by administration of salbutamol and congestion on heart means this has been repaired with treatment of herbal mixture. Liver was almost normal in all groups. But in curative group liver was slightly necrosed that means this group cures disease by post administration of herbal mixture. This may be due to high plant dose 500 mg/ kg or some unknown factors. So further studies should be done on this herbal mixture with lessen or modified amount of dose.

## Conclusions

Herbal mixture shows cardio protective and antilipidemic activity. Some studies were reported the cardio protective activity of these individual plants. However, no pervious data is available on cardio protective and antilipidemic effect of herbal mixture of these three indigenous medicinal plants. It has been first time reported in this study that mixture of *Coriandrum sativum, Cactus grandiflorus* and *Piper nigrum* showed cardio protective and hypolipidemic effect.

## Electronic supplementary material

Below is the link to the electronic supplementary material.

Supplementary Material 1
