# Supplementary figures and images for: Investigation of phytotherapeutic potential of herbal mixtures and their effects on salbutamol induced cardiotoxicity and hyperlipidemia in rabbits
Source: Bot Stud. 2023 Jul 19;64:23. doi: 10.1186/s40529-023-00394-9 (PMC10356716; doi:10.1186/s40529-023-00394-9)

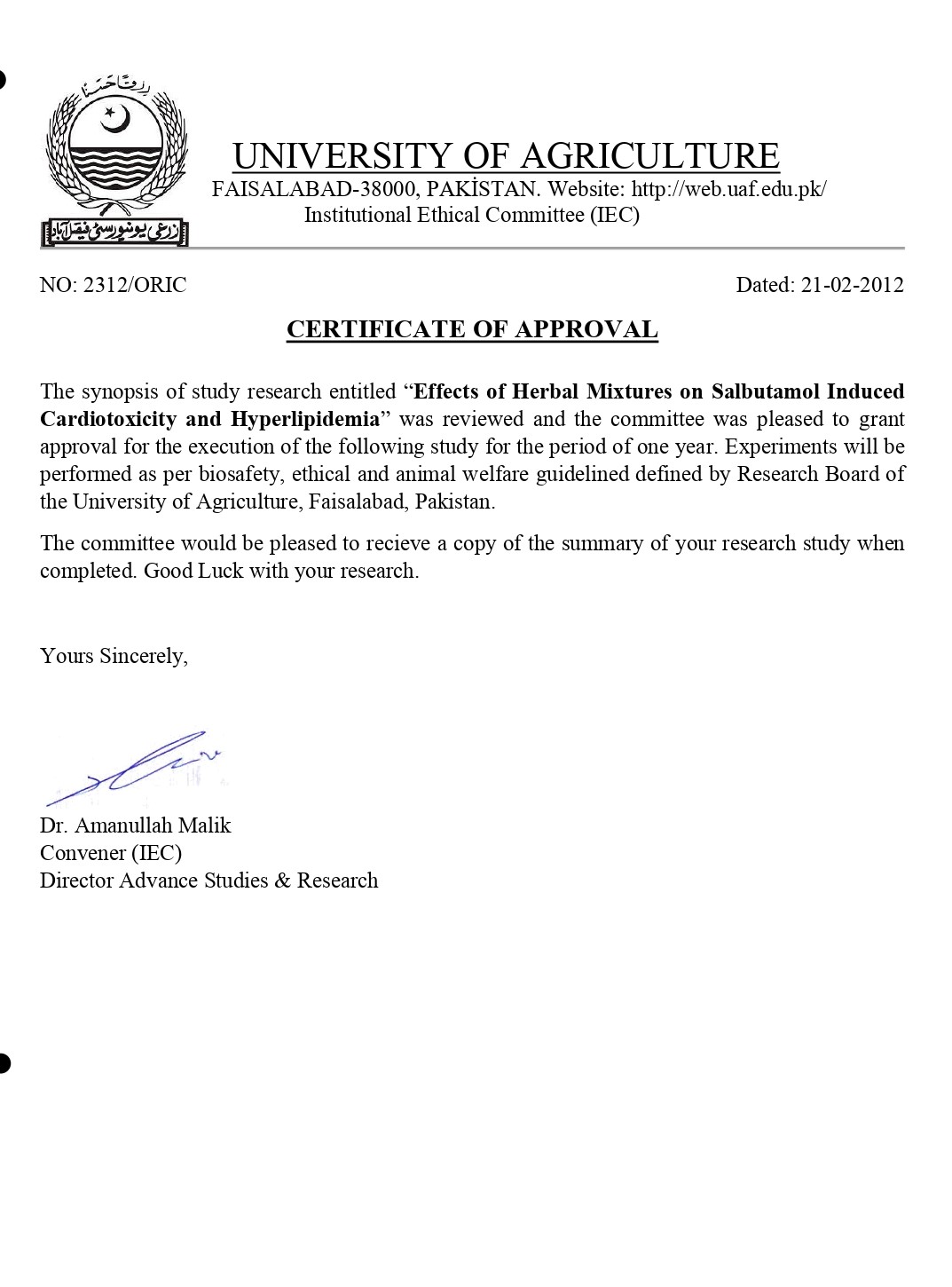

Supplement: Supplementary file 1 — Supplementary Material 1 [file 40529_2023_394_MOESM1_ESM.jpg]
